# A Rare Case of Dyke-Davidoff-Masson Syndrome in an Adolescent Female

**DOI:** 10.7759/cureus.56377

**Published:** 2024-03-18

**Authors:** Ankita Sachdev, Sourya Acharya, Harshita J, Shreyash Huse

**Affiliations:** 1 Department of Medicine, Jawaharlal Nehru Medical College, Datta Meghe Institute of Higher Education and Research, Wardha, IND

**Keywords:** rare disease, status epileptics, hyperpneumatization, perinatal injury, hypoxic brain

## Abstract

The Dyke-Davidoff-Masson syndrome (DDMS) is an uncommon neurological disorder whose prevalence is not yet known. There have only been 21 adult manifestations of this rare brain disorder, out of around 100 cases previously documented. Diagnosis is challenging because of the complexity of radiological findings and clinical symptoms, which include ventricle dilation, hypertrophy of the cranial bones, increased pneumatization of the sinuses, and cerebral hemisphere atrophy. It can be inherited or acquired from infections, brain hemorrhage, and hypoxia during pregnancy. Usually, neuroimaging is used to diagnose it. This case study reports the case of a 17-year-old girl who had complex partial seizures at the age of 17 and right-side paralysis since she was three years old.

## Introduction

The radiologically recognized condition known as Dyke-Davidoff-Masson syndrome, also known as DDMS, is typified as treatment-resistant epilepsy, mental retardation, contralateral hemiparesis, thickening of the skull bone linked to cerebral atrophy, atrophy of the cerebellum and basal ganglia, and hyperpneumatization of the frontal and paranasal sinuses on imaging [1]. Other symptoms that some people may exhibit include ear abnormalities and neuropsychiatric problems. The two main kinds of DDMS etiologies are acquired and congenital [2]. Two congenital causes of DDMS include cerebral hemisphere hypoperfusion and intrauterine vascular injury. The most common acquired causes are ischemic stroke, radiation, cerebral hemorrhage, postictal cerebral hemiatrophy, periventricular leukomalacia, and prolonged febrile convulsions [3].

The left cerebral hemisphere is the primary site of involvement for DDMS, which typically affects men. The period of the neurological injury determines the age of presentation; most instances are identified in childhood or adolescence because these are the most common periods for clinical signs to occur [4]. There is a large range of clinical manifestations for DDMS. The illness typically manifests as contralateral hemiparesis, varying degrees of facial asymmetry, convulsions, mental impairment, and behavioral abnormalities [2].

## Case presentation

A 17-year-old girl with two episodes of seizures and impaired sensorium was brought to the hospital. After the episode, she was unresponsive to any verbal, tactile, or painful stimulation for 10 minutes due to post-ictal disorientation. The patient had no memory of the incident. Her mother provided a thorough history. She was born into a non-consanguineous marriage and was delivered institutionally through a full-term normal delivery. The patient experienced acute, persistent weakness in the right side of her body at the age of three. Power was 1/5 on the right side and 5/5 on the left side of the body, along with a deviation of the angle of the mouth on the left side. The patient underwent acupuncture therapy for four months in addition to being taken to an Ayurvedic facility. She was bedridden for the first month, but over the next four months, she improved. After a year, she could walk with assistance, and by the time she was five years old, she could use her upper limb and walk on her own.

**Figure 1:**
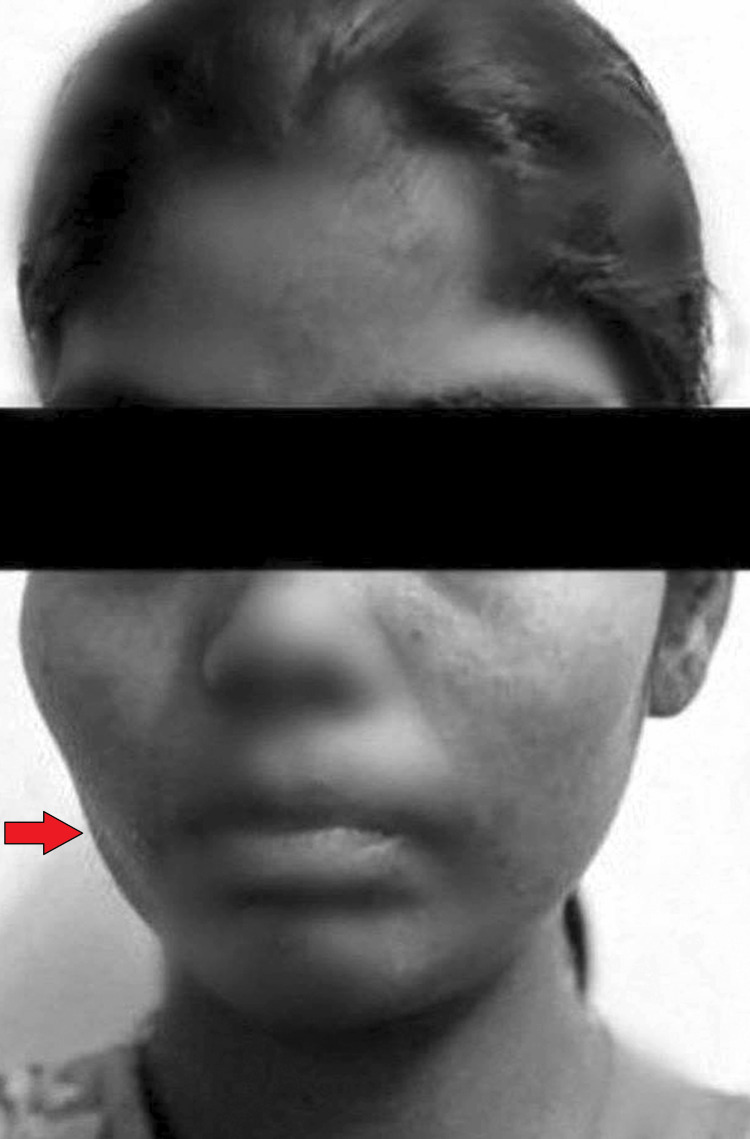
Clinical image of a patient with Dyke-Davidoff-Masson syndrome shows facial asymmetry (black arrow) Image Credit: Author

**Figure 2:**
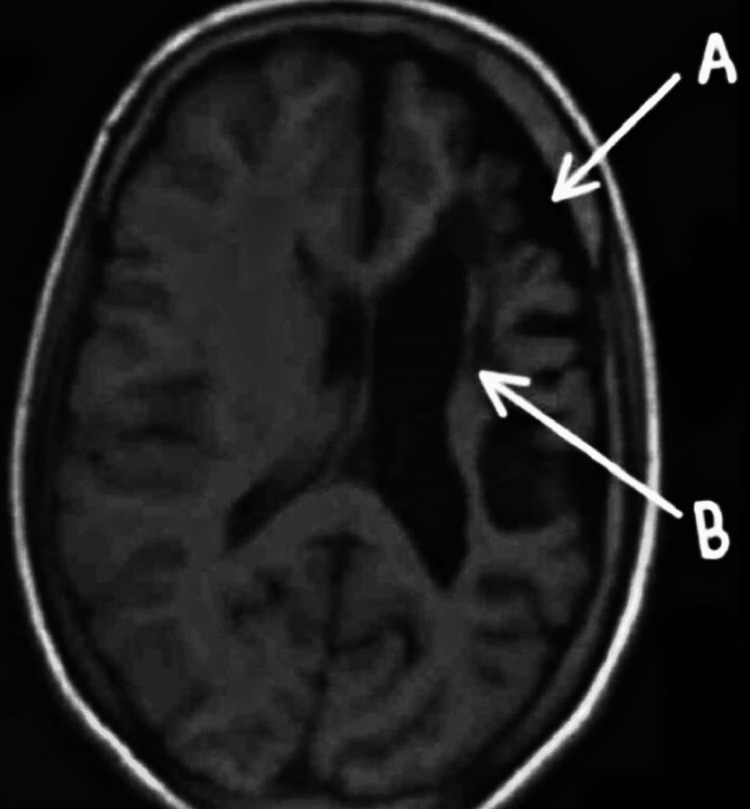
A: T1-weighted axial MRI reveals atrophy in the left frontoparietal and temporal areas of the brain. B: Dilatation of the left lateral ventricle

**Figure 3:**
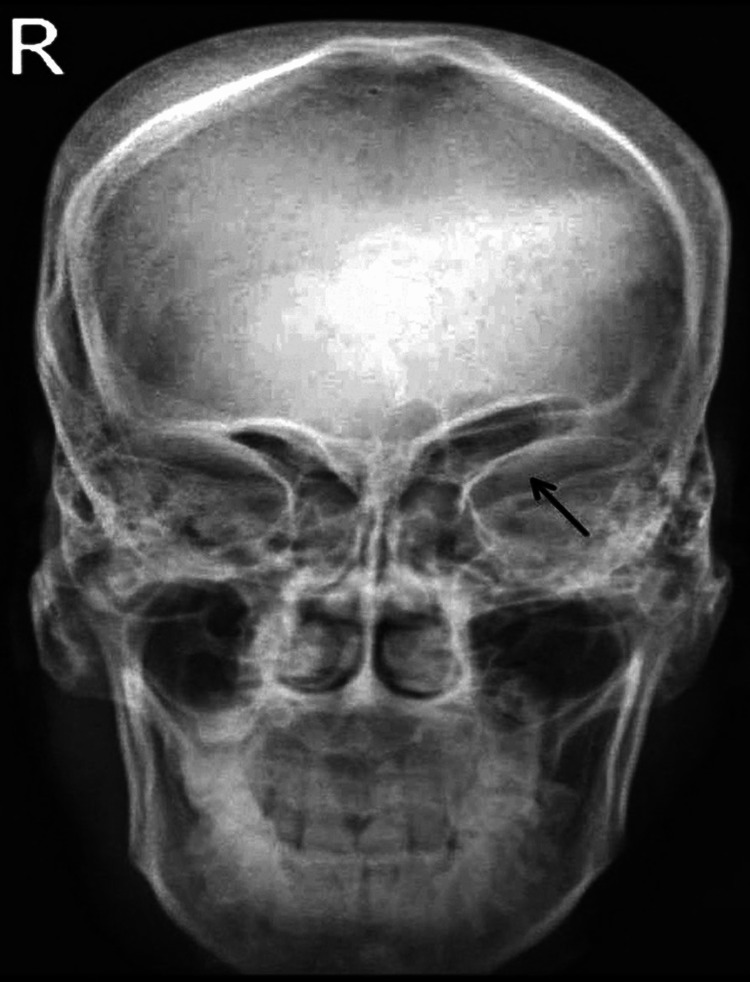
X-ray of the skull reveals hyperpneumatization of the left sinus (black arrow)

Upon assessment, she was conscious and oriented, and her blood pressure, oxygen saturation, pulse, and respiration rate were all within normal limits. Figure 1 suggests her facial asymmetry. She had hyperreflexia, power 4/5, decreased muscle mass, and increased tone in her right upper and lower limbs, and she also had an extensor plantar reflex on the right side. Figure 2 shows that gliotic areas involving the left frontoparietal and temporal regions cause volume loss with ex vacuo dilatation of the lateral ventricle on the left side and a paradoxical shift of the midline towards the left side on radiological evaluation. These areas are likely the result of previous insults such as perinatal hypoxic or ischemic injury. She showed a right hip external rotation, a pelvic drop to the right side, and an enhanced pneumatization of the sinus, as seen in Figure 3. The right shoulder was drooping, and the upper limb was hanging. Her sensory testing, cortical sensation, and bowel and bladder functions were all normal. Normal blood cells, an unremarkable fasting lipid profile, a negative result for protein C and S deficiency, and normal kidney and liver function tests were all confirmed by the laboratory study.

## Discussion

The given name of this rare illness is credited to scientists Masson, Davidoff, and Dyke. In 1933, they first documented the condition [2]. Seizures, hemiparesis, mental disability, and facial asymmetry were reviewed in nine patients with plain skull radiography [5]. A typical imaging finding is severe or subtotal cerebral hemiatrophy. However, unilateral localised atrophy may rarely be seen in the thalamic, pontine, cerebellar, parahippocampal, and cerebral peduncles [6-8]. Brain imaging can also reveal features such as ipsilateral hypertrophy of the cranial bone, a raised temporal bone, greater pneumatization of the sinuses, calvarial thickening, clearly visible cerebral sulci, larger lateral ventricles, and cisternal space [1-3].

Clinical features of DDMS include learning impairments, mental retardation, focal seizures or generalised seizures, contralateral hemiparesis, and the upper motor neuron form of facial palsy [5]. Any part of the brain could be affected, though studies have indicated that left-sided involvement and male gender are more common [6]. In DDMS, there are two types of cerebral hemiatrophy that are commonly seen. The infantile or congenital variety, which is caused by unilateral cerebral circulation defects such as coarctation of the arch of the aorta, affects individuals during the prenatal or infancy periods of life. Multiple factors, including birth trauma, protracted seizures, malignancy, infection, and bleeding, might result in the acquired type [7,8].

The classical MRI changes associated with this syndrome do not appear until after the age of three unless there has been a brain trauma from one of the many possible causes [9]. It is possible that recurrent ischemic episodes from different sources are responsible for cerebral atrophy and its gradual progression [5]. Cerebral atrophy is facilitated by these episodes because they reduce the release of neurotrophic substances produced by the brain [10]. The growing human brain exerts external stress on the bones of the head surface during the first few weeks of life, causing a child's head to achieve its maximum size at 50% of an adult's size during the final days of early childhood and a third of an adult's size by the time the kid is three years old. Thus, extra brain-overlying structures only start to expand inward when injury to the brain occurs before the age of three. This causes the illness's characteristic diploic gaps to deepen, the sinuses to grow, and the orbital roof to rise [7,9].

The patient's age at presentation, facial asymmetry, seizures, hemiatrophy, large ventricles, and calvarial abnormalities in the brain's imaging all support the diagnosis in this particular case. These findings also aid in distinguishing it from Sturge-Weber syndrome and Rasmussen's encephalitis. Better results are seen if hemiparesis develops beyond two years of age and there are no protracted or frequent seizures [11]. Appropriate anticonvulsants should be used to treat patients who have refractory seizures [4]. Additionally, speech, occupational, and physical therapy can all have a significant impact on the patient's prognosis. To enhance speech quality, speech therapy should incorporate activities that require the patient to articulate and use proper vocal tones. Exercises used in occupational therapy will help patients overcome challenges related to their physical, cognitive, and developmental needs, as well as aid in their recovery and upkeep of everyday activities [3,4]. With an 85% success rate, surgical removal of half of the cerebral hemisphere is the recommended course of treatment for patients with intractable, incapacitating seizures and hemiparesis [8].

## Conclusions

The given case of DDMS draws attention to the difficulties in diagnosing and treating this uncommon ailment early on. Due to its rarity, the diagnosis is often missed by physicians. Therefore, we are presenting this case in an effort to close any knowledge gaps about DDMS. Imaging modalities such as CT and MRI can be utilized to identify the key imaging characteristics linked to this illness. For proper patient management, knowledge of imaging characteristics, risk factors, and clinical presentation is essential. Although considered a childhood condition, it can present as an adult even in the absence of a relevant pediatric background. The primary focus of treatment is symptomatic care, while in some circumstances, surgery is recommended as a last resort.
